# Rapid loss of seed viability in *ex situ* conserved wheat and barley at 4°C as compared to −20°C storage

**DOI:** 10.1093/conphys/coy033

**Published:** 2018-06-25

**Authors:** Rob van Treuren, Noor Bas, Jan Kodde, Steven P C Groot, Chris Kik

**Affiliations:** 1 Centre for Genetic Resources, Wageningen Plant Research, Wageningen, the Netherlands; 2 Department of Bioscience, Wageningen Plant Research, Wageningen, the Netherlands

**Keywords:** Cereals, genebanks, germination, seed longevity, seed viability, storage temperature

## Abstract

Genebanks aim to optimize their storage conditions in order to postpone seed ageing as long as possible. As most genebanks have a relatively short life history, empirical data about seed longevity during *ex situ* storage are almost absent. Based on seed characteristics, theoretical predictions indicate that cereal seeds can be stored without substantial loss of viability for time periods exceeding 100 years, even under temperatures of a few degrees above zero. Here we present the results of a germination study in wheat and barley, comparing genebank seed samples maintained at different temperatures for 23–33 years. Wheat and barley seed samples stored at −20°C showed a mean germination of 94% and 90%, respectively, indicating no loss of the initial viability determined for the accessions prior to introduction in the collection. Seed samples maintained at 4°C showed a mean germination of 62% for wheat and 75% for barley. In addition to the observed loss of viability, the 4°C samples also showed a loss in vigour as the time period to reach their final germination was about twice as long compared to the −20°C samples. A subset of the wheat accessions tested in 2011 were retested in 2017, showing further reduction in mean germination to 35% for the 4°C samples, while the −20°C samples remained stable at 95%. Several 4°C samples were even close to a complete loss of viability. Considering that wheat and barley are generally regarded as good maintainers, the rapid loss of seed viability observed in the present study indicates that the *ex situ* seed storage of genetic resources at 4°C should be treated with caution by genebanks, particularly when used for long-term conservation.

## Teaser

Wheat and barley seeds conserved for two to three decades under genebank conditions showed severe losses of viability when stored at 4°C, while no reductions were observed under −20°C. These findings should concern genebank curators, especially when suboptimal temperatures are used for long-term seed storage.

## Introduction

Genebanks manage seed collections for the conservation and utilization of plant genetic resources. To avoid the frequent rejuvenation of accessions, which is laborious and costly and may cause loss of genetic integrity, genebanks aim to prolong seed longevity as much as possible (Engels and Visser, 2003). As the majority of genebanks were established in the 1960s/1970s and because seeds have a high life expectancy under genebank storage conditions, it is largely unknown how long seeds can be stored under *ex situ* conditions without significant loss of viability and how monitoring intervals can be optimized (Walters *et al.*, 2005; Hay *et al.*, 2013; Van Treuren *et al.*, 2013; Ellis *et al.*, 2017). Low seed moisture content and storage temperature are generally considered as important criteria to maintain the viability of orthodox seeds (Ellis and Roberts, 1980a; 1980b), while anoxic conditions have also been recommended to optimize seed longevity (Ellis and Hong, 2007a; Groot *et al.*, 2015).

Standards for genebank management recommend a temperature of −18 ± 3°C for long-term storage of the base collection and 5–10°C for medium-term storage of the active collection (FAO, 2013). The seed information database of the Royal Botanic Gardens Kew (2017) offers a tool to estimate the longevity of seeds, based on the seed viability equation of Ellis and Roberts (1980a). Using experimentally derived species specific constants (Ellis *et al.*, 1990), the predicted storage time for wheat (*Triticum aestivum*) seeds to fall from 95% to 80% viability is 424 years under −20°C and 105 years under 4°C when drying conditions of 15°C and 15% RH and an seed oil content of 2.2% are assumed. Assuming similar drying conditions and 2.0% seed oil content, the predictions are 573 years at −20°C and 142 years at 4°C for barley (*Hordeum vulgare*) using the species specific constants of Ellis and Roberts (1980b) and Dickie *et al.* (1990). Therefore, wheat and barley are generally considered good maintainers (e.g. Nagel *et al.*, 2009).

The Centre for Genetic Resources, the Netherlands (CGN) maintains the Dutch national collection of plant genetic resources, which includes nearly 5000 accessions of wheat and nearly 2700 of barley (CGN, 2017). Prior to packaging, seed samples of the CGN collection are dried at 15°C and 15% RH for 2 months in a controlled environment. According to genebank standards the initial germination value should exceed 85% for cultivated crop species, while lower values are accepted for materials that do not normally reach high levels of germination, such as crop wild relatives (FAO, 2013). Based on a sample size of 200 seeds, threshold values of 80% and 60% are used by CGN for cultivated and wild materials, respectively. Seed samples fulfilling these requirements are packed in three-layered aluminium foil bags that are vacuum-sealed before storage. Seed bags intended for regeneration and germination testing, as well as a large spare seed quantity, are stored at −20°C. For crops with a high life expectancy, such as wheat and barley, seed bags intended for distribution to users are stored at 4°C because handling procedures are more preferable at this temperature. Since the establishment of CGN in 1985 until the end of 2016, ~11 000 seed samples of wheat and 3500 of barley have been distributed for utilization.

To check whether seeds maintain sufficient viability during storage, the germination ability of CGN samples stored at −20°C is periodically monitored (Van Treuren *et al.*, 2013). When germination has dropped below the threshold value, the accession is rejuvenated to produce next generation seed lots with sufficient viability. However, the germination ability of user samples stored at 4°C is not monitored by CGN. Feedback from the user community is the main source of information on seed viability for these samples. In 2007, feedback was received regarding 355 distributed wheat accessions, reporting 88 seed samples (25%) with a germination percentage below 60%. In the same year another user reported the absence of viability for 24 accessions (55%) out of 44 distributed wheat accessions. A small-scale germination experiment with five wheat accessions confirmed the reduced viability of the samples stored at 4°C. Here we report a comprehensive viability study on wheat and barley comparing the germination ability between samples stored at 4°C and at −20°C. The main goal of the study was to investigate whether the genebank management procedures need optimization in order to provide better services to our user community.

## Materials and methods

### Study material

The seeds used in the present study had been stored at CGN in laminated aluminium foil bags, consisting of an inner layer of 75 μm polyethylene, a middle layer of 12 μm aluminium and an outer layer of 12 μm polyester for printing. As older materials are more likely to have experienced greater seed ageing, accessions were selected that had been regenerated before 1990. Five regeneration years were selected per crop, namely 1978–1979 and 1984–1986 for wheat and 1985–1989 for barley (Supplementary Table 1). These regeneration years were selected also because of the large number of accessions regenerated for these crops in those years. For each of the crops, 25 accessions were randomly chosen per regeneration year, provided that seed samples were available that had been stored at 4°C since the introduction in the CGN collection. Seeds of these samples and those stored at −20°C originated from the same seed batch and had experienced identical handling procedures and environmental conditions during genebank management. Thus, storage temperature is the single variable distinguishing the different samples of the study accessions. The study accessions of wheat consisted of 62 cultivars, 54 landraces and 9 research materials, all belonging to *T. aestivum*. For barley 34 cultivars, 75 landraces, 5 research materials and 11 accessions of unknown population type of *H. vulgare* were used in the study. Using the accession numbers presented in Supplementary Table 1, more information about the study materials can be obtained from the CGN website (CGN, 2017).

### Germination testing

All germination tests were carried out with 100 seeds per sample. The two differently stored samples of an accession were sown in a single tray with compost. Trays were randomly distributed on work tables in a greenhouse facility of Wageningen Plant Research. Wheat samples sown in July 2011 experienced an average temperature of 20°C during daytime and 15°C during the night. Barley samples were sown in January 2012 in a heated greenhouse and were exposed to 16 h of assimilation light (natural conditions and additional light from Son-T, 90 μmol), while the average temperature was 17°C during daytime and 16°C during the night.

Germination was scored according to the methods described by the International Seed Testing Association (Don, 2003). Germination recording started 6–7 days after sowing. Plants without abnormalities which had grown to a height of at least 10 cm were recorded as healthy seedlings. Plants that showed chlorophyll deficiency during early growth but later developed into vital plants were also considered as healthy seedlings.

To investigate temporal developments in viability, a subset of 25 wheat accessions were retested in 2017 (Supplementary Table 1). Five accessions per regeneration year were selected on the basis of the germination results of the 4°C samples tested in 2011, representing the worst two, the best two and an intermediate. Seeds were sown in July in an unheated greenhouse followed by the same test procedures as used for the material examined in 2011. The samples were exposed to an average temperature of 23°C during daytime and 17°C during the night.

### Analysis of germination data

For each sample the fraction germination was calculated as the observed number of healthy seedlings divided by the number of tested seeds. Differences in germination fraction between seed samples stored at different temperatures were tested for significance using a two-tailed *z*-test, where *z* = (*p*_1_ − *p*_2_)/σ_*p*1_ − _*p*2_ and *p*_1_ and *p*_2_ denote the fraction germination of the samples. The standard deviation σ_*p*1_ − _*p*2_ is given by sqrt{*p* * (1 − *p*) * [(1/*n*_1_) + (1/*n*_2_)]}, where *p* = (*n*_1_ * *p*_1_ + *n*_2_ * *p*_2_)/(*n*_1_ + *n*_2_) and *n*_1_ and*n*_2_ denote the sample sizes. A significance level of 0.05 was used to test the standardized variable *z*.

In graphical representations of mean germination fractions, the variability of the data was indicated by error bars, calculated as the standard deviation of the mean value divided by the square root of the sample size. Viability test results were presented in relation to initial germination values, determined prior to the introduction of an accession in the collection and storage of the seed samples in the genebank facility. These tests were carried out by ISTA-certified agencies, using rolled paper towels and controlled laboratory conditions.

### Examination of seed equilibrium relative humidity

The water activity (*a*_w_) of a representative group of 10 wheat and 10 barley samples was examined in 2018 to investigate whether the seeds had maintained the moisture level during storage at −20°C or 4°C after drying at 15°C and 15% RH. These measurements were carried out on a subset of the materials used for germination testing as seed bags stored at 4°C were no longer available for several accessions. To investigate whether differences in germination were associated with differences in seed moisture content, the accessions selected for *a*_w_ measurements covered both low and high germination test results for each of the five examined regeneration years. Measurements of *a*_w_ were performed using a HygroLab Bench Top Indicator version 4 (Rotronic AG, Bassersdorf, Switzerland). The probes were calibrated at 20°C with drying beads (*a*_w_ = 0.0), moisturized ZnBr salt (*a*_w_ = 0.08), moisturized CaCl_2_ salt (*a*_w_ = 0.32), moisturized NaCl salt (*a*_w_ = 0.75), moisturized KCl salt (*a*_w_ = 0.85) and pure water (*a*_w_ = 1.0). The water activity station or probe of the HygroLab was placed on top of the sample holder that was at least half filled with seeds. The resulting closed system was allowed to reach equilibrium for at least 2 h, after which the indicated *a*_w_ was recorded. Based on the *a*_w_ value at 20°C, seed equilibrium relative humidity at 15°C was calculated using the seed viability equation of Cromarty *et al.* (1982). The *a*_w_ was converted to equilibrium relative humidity (eRH) considering an *a*_w_ of 0.0 and *a*_w_ of 1.0 as 0% and 100% eRH, respectively. Storage behaviour based on the eRH value at 15°C was predicted using the viability calculation tool of the Royal Botanic Gardens Kew (2017). Seed moisture content was determined for a subset of the selected samples using the high constant temperature oven method (ISTA, 2018). Seeds were dried at 130°C for 4 h, and the resulting moisture content is presented on a fresh weight basis.

## Results

**Figure 1:**
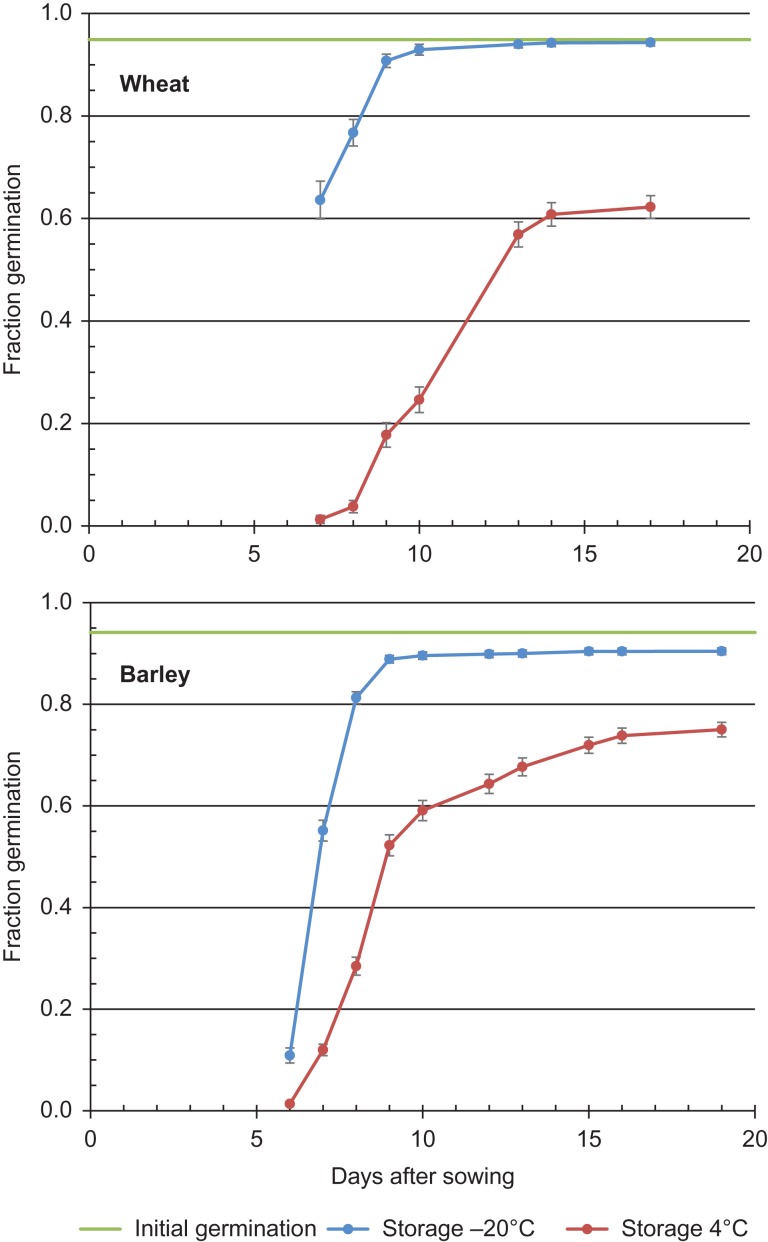
Progress of germination of seed samples of 125 wheat and 125 barley accessions maintained for 25–33 years and 23–27 years, respectively, under two different temperatures during genebank storage. The green line represents the mean initial germination of the tested accessions prior to storage.

**Figure 2:**
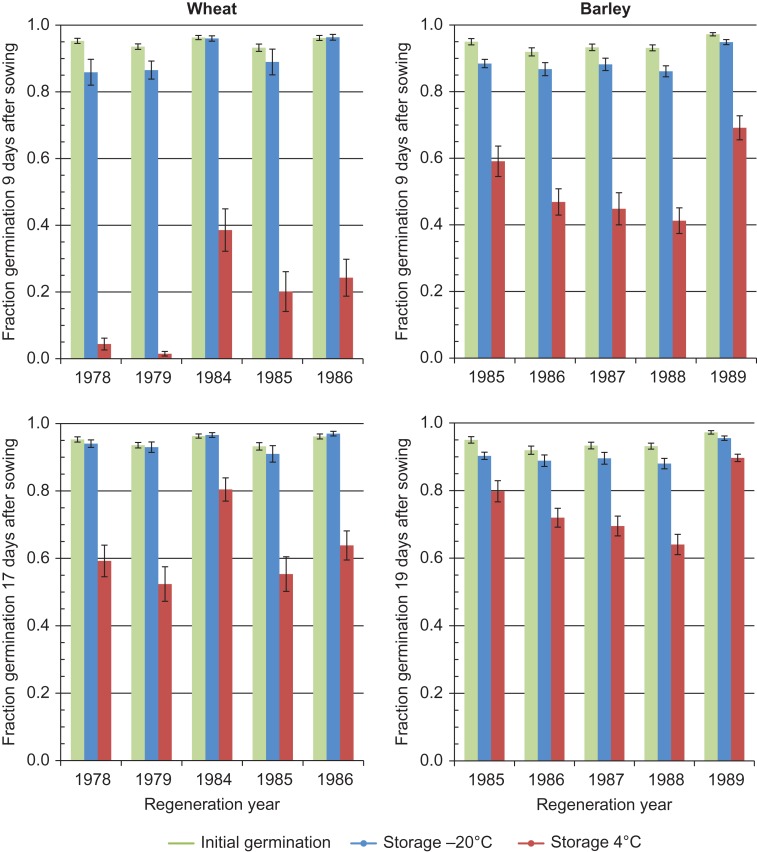
Mean germination of seed samples of wheat and barley accessions maintained for 25–33 years and 23–27 years, respectively, under two different temperatures during genebank storage, presented separately for different regeneration years. Tests were performed in 2011 for wheat and in 2012 for barley. Germination is shown for 9 days after sowing and for the final recording day, which was 17 days after sowing for wheat and 19 days for barley. Mean initial germination prior to storage is indicated by green bars.

For 109 out of the 125 wheat accessions (87%) tested in 2011, the seed samples stored at 4°C showed a significantly lower final germination than those maintained at −20°C. Only in the case of accession CGN04075 was a significantly higher germination observed for the 4°C sample, namely 97% versus 42% for the −20°C sample. For barley, 76 out of the 125 tested accessions (61%) showed a significantly lower final germination for the 4°C samples, while 49 showed no significant difference. The mean final germination in wheat was 62% for the 4°C samples and 94% for the −20°C samples, the latter being close to the mean initial germination of 95% determined prior to the introduction of the accessions in the collection. Similar results were observed for barley with mean final germination values of 75% and 90%, respectively, and a mean initial germination of 94% (Fig. 1). The samples stored at different temperature did not only differ in germination percentage but also in vigour as the −20°C samples approached their final germination by 9 days after sowing, while this lasted several days longer for the 4°C samples (Fig. 1). These differences in germination speed were not confined to specific regeneration years, although the magnitude of the effect was found to vary among the years (Fig. 2). For example, the 4°C samples of the wheat accessions regenerated in 1978 and 1979 showed less than 5% germination on average after 9 days, while the wheat accessions regenerated in 1984 had reached nearly 40% by that time. Also the final mean germination of the 4°C samples differed among regeneration years, ranging from 52% for 1979 to 80% for 1984 in wheat, and from 64% for 1988 to 90% for 1989 in barley. Although ageing effects can be expected to be more severe for older seeds, the final germination of the 4°C seed samples was not found to decrease with longer time since regeneration (Fig. 2).

**Figure 3:**
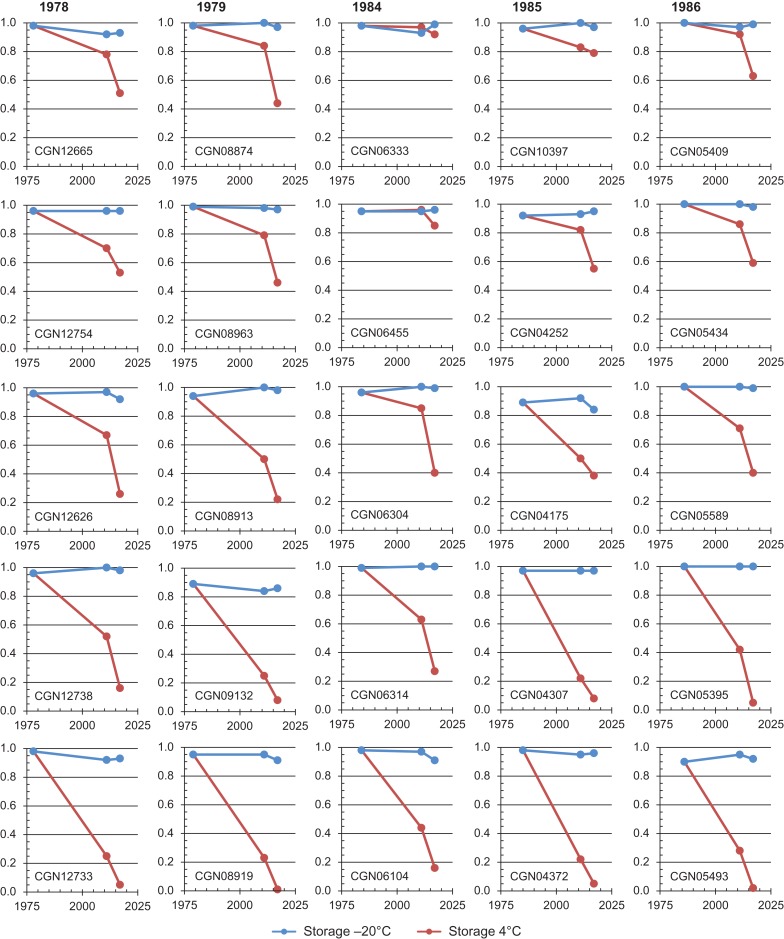
Temporal changes in germination of seed samples of 25 retested wheat accessions regenerated in five different years and maintained under two different temperatures during genebank storage. The fraction germination (*y*-axis) is presented for different testing years (*x*-axis) and includes the initial germination of the accessions prior to storage and the results of the germination tests performed in 2011 and 2017. Accession codes are presented in the bottom left corner of each graph and regeneration years are denoted at the top of each set of 5 graphs.

Further reductions in final germination of the 4°C seed samples were observed for each of the 25 wheat accessions retested in 2017, whereas the germination rate of the −20°C samples remained relatively stable in comparison with the test results of 2011 and the initial viability of the accessions (Fig. 3). Apart from CGN06333 and CGN06455, both regenerated in 1984, the germination rate of the 4°C seed samples of all study accessions dropped below CGN’s threshold level of 80%. Several of these accessions were even found to be close to the complete loss of viability, such as observed for the 4°C seed samples of CGN08919 regenerated in 1979 and CGN05493 regenerated in 1986.

Seeds that are dried under conditions practised by CGN are expected to show an eRH of ~15% and a moisture content of about 6% for wheat and barley. In general, eRH values were found to be lower for the samples stored for several decades at −20°C as compared to 4°C storage at CGN (Supplementary Table 2). The eRH of seed samples stored at 4°C ranged from 14.9% to 21.1% for wheat and from 15.0% to 25.2% for barley (Table 1). Despite these rather wide ranges, observed eRH was found to be a poor predictor of the effects on viability. In all cases where a reduction in germination was observed, the effect was more severe than could be expected based on the measured eRH. For example, the germination of barley accession 1692 dropped from 96% to 29% during 25 years of storage. Based on the measured eRH of 15% a small reduction to 95% germination is expected within a period of 25 years, while the expected time to drop to 29% is 407 years (Table 1). Seed moisture content determined for a limited number of seed samples ranged from 5.9% to 6.2% for wheat and from 5.5% to 7.0% for barley when restricted to seed bags that still showed complete vacuum after storage (Supplementary Table 2). Thus, compared to eRH, observed values of seed moisture content were more in line with expectations based on CGN’s drying conditions. Our findings suggest that seed moisture levels do not account for the rapid reduction in germination observed for wheat and barley seeds under 4°C storage conditions.

**Table 1:** Equilibrium relative humidity (eRH) at 15°C determined in 2018 for wheat and barley seed samples^a^ stored at CGN at 4°C

Accession	eRH	G0	G1_obs_	G1_exp_	Yr_obs_	Yr_exp_
Wheat						
5752	14.9	0.99	0.81	0.98	25	190
9267	16.0	0.94	0.36	0.90	32	207
4123	16.8	0.96	0.29	0.93	26	208
8539	17.3	0.97	0.77	0.93	32	94
6027	18.3	0.96	0.86	0.91	27	46
12 722	23.5	0.98	0.77	0.86	33	44
6455	21.1	0.95	0.96	0.85	27	–
Barley						
1692	15.0	0.96	0.29	0.95	25	407
2613	15.5	0.95	0.42	0.93	27	299
2068	16.3	0.99	0.98	0.98	26	37
113	20.1	0.98	0.52	0.96	26	157
859	20.7	0.97	0.90	0.94	25	44
11 302	22.5	0.82	0.32	0.68	24	75
2698	16.1	0.99	0.99	0.98	24	–
1806	25.1	0.95	0.97	0.86	23	–
157	25.2	0.98	0.99	0.92	27	–

‘G0’ indicates the fraction germination prior to storage, ‘G1_obs_’ the fraction germination examined for wheat in 2011 and for barley in 2012 and ‘G1_exp_’ the expected fraction germination based on the equilibrium relative humidity. ‘Yr_obs_’ and ‘Yr_exp_’ denote the observed and expected number of years for germination to drop from G0 to G1_obs._

^a^Only samples with complete vacuum are presented.

## Discussion

Seed samples of wheat and barley maintained at the genebank facilities of CGN showed a rapid loss of viability at 4°C in comparison with storage at −20°C. Whereas the germination of accessions was found to remain stable under storage at −20°C, a reduction from 95 to 62% in 25–33 years for wheat and from 94% to 75% in 23–27 years for barley was observed at 4°C. According to Kew’s seed viability calculator, such losses in germination at 4°C for seeds with ~2% oil content that are dried at 15°C and 15% RH can be expected after 175 and 155 years, respectively (Royal Botanic Gardens Kew, 2017). Wheat and barley samples of CGN also deteriorated more rapidly compared to published predictions of the effect of the seed storage environment on longevity (Ellis and Hong, 2007b). The observed results cannot be attributed to strongly fluctuating conditions during storage as CGN’s facilities are monitored and registered data show only occasional fluctuations in temperature in the 4°C facility, which never exceeded a 1°C deviation.

A loss of viability in aged seed lots is generally accompanied by reduced seed vigour (e.g. Zurek, 1999). This effect was also observed in the present study as, compared to the −20°C samples, the 4°C samples showed lower germination speed and higher levels of chlorophyll deficiency during early growth. Only in the case of wheat accession CGN04075 a significantly higher viability was observed for the 4°C sample in comparison to the −20°C sample. Although this single atypical result was perhaps due to the switching of these two samples during seed sowing, the data for this accession were included in all analyses as these had only negligible effects on the main findings of the study.

The loss of seed viability as a function of time usually follows a sigmoid pattern, the characteristics of the curve being variable among species and considered to be mainly dependent on storage temperature and seed moisture content (Ellis, 1991; Nagel and Börner, 2010). However, seed production conditions, post-harvesting procedures prior to storage in the genebank facility and the extent to which seeds are exposed to oxygen are considered important additional factors influencing seed ageing (Hay and Probert, 1995; Ellis and Hong, 2007a; Groot *et al.*, 2015; Yadav and Ellis, 2016). The fact that such variables are not taken into account in Kew’s seed viability calculator could potentially cause empirical results on seed viability to differ markedly from theoretical predictions. However, it is considered unlikely that these factors underlie the results observed in the present study as seed management procedures at CGN largely follow agreed international standards (FAO, 2013), while the time period between seed production and final storage has been minimized as much as possible and seeds are maintained under vacuum, creating reduced oxygen levels (Groot *et al.*, 2015). Part of the accessions examined in the present study originated from working collections and experienced different management procedures prior to introduction to the collection. For example, cereal seed samples from the former Foundation for Agricultural Plant Breeding (SVP) were maintained at 3°C and 30% RH in paper bags prior to genebank storage (Van Soest and Dijkstra, 1984), while those from the former Institute for Plant Breeding of the Agricultural University Wageningen (LHIVP) were first regenerated by CGN. In the present study the 4°C wheat samples with a SVP background (*n* = 51) and those with a LHIVP history (*n* = 72) showed a mean final germination of 57% and 66%, respectively. Thus, notwithstanding the historical differences in the quality of the storage conditions, a strong reduction in viability was observed for both type of samples. As seed ageing was also observed for accessions regenerated after the establishment of CGN, it is considered unlikely that suboptimal handling procedures are a main cause of the rapid loss of viability under 4°C storage.

The rate of seed viability loss can be expected to increase with storage time, as shown by the wheat samples retested in 2017. However, no consistent relationship between time since regeneration and loss of viability was observed in the present study. Small-scale viability experiments on more recently produced samples were performed in 2014 using 20 wheat and 20 barley accessions regenerated by CGN between 2001 and 2003. The wheat accessions with a mean initial germination of 91% showed a mean germination of 90% for the samples stored at −20°C and 84% for those stored at 4°C, while the barley accessions with a mean initial germination of 92% showed a mean germination of 90% and 83% for the −20°C and 4°C samples, respectively (our unpublished work). These data suggest that seed deterioration at 4°C starts already in the early stages of seed storage. More comprehensive testing of time series should elucidate how exactly seed viability can be expected to decrease in time under storage at 4°C.

The species specific constants for wheat and barley have been determined using single seed lots, thus from a single variety and seed production condition. For several species, including wheat and barley, considerable genetic variation in seed longevity has been found (Schwember and Bradford, 2010; Nagel *et al.*, 2011, 2016; Arif *et al.*, 2017). Also in our experiments we observed that some accessions, such as CGN06333 and CGN06455 of wheat, did not seem to have suffered from storage at 4°C as they maintained their high germination rate even when tested again in 2017. These accessions were regenerated in a larger batch of accessions. Checking of the regeneration and seed handling records did not reveal any deviations for these accessions from standard procedures. Both CGN06333 and CGN06455 were regenerated in 1984, which in general revealed higher germination rates compared with the other examined regeneration years. Differences in seed viability between regeneration years were also observed for barley. These differences may possibly have been due to unintentional variation in regeneration conditions among years or to variation in characteristics of the material regenerated in different years. However, potential causes of variation in seed ageing between regeneration years could not be substantiated by available accession data.

Germination experiments performed on the same materials may show variation in test results (Van Hintum and van Treuren, 2012), and higher levels of variability may be expected when different tests procedures are used. The procedures used for determining initial germination rates differed from those used in the present study. It is considered unlikely that the reduction of viability observed for 4°C samples was due to variation in test procedures as the −20°C samples showed stability in seed viability in comparison to the initial tests. In fact, the reported losses of viability for 4°C samples should rather be considered underestimations as according to ISTA procedures the final germination for wheat is recorded on day-8 and for barley on day-7 (Don, 2003), showing only 4% and 12.0% germination, respectively.

The rapid loss of viability observed for wheat and barley seeds at 4°C resembles the results of an experimental study performed by Stanwood (Walters *et al.*, 2005). In the study of Stanwood, a reduction in germination from 95% to 26% was observed for seeds of wheat cultivars (*n* = 3) that had been stored for 40 years at 5°C. Barley cultivars (*n* = 3) maintained at 5°C showed a reduction from 97% to 73% seed germination in 26 years in that study. During the experiments of Stanwood, seeds were most likely exposed to atmospheric oxygen as storage occurred in envelopes, plastic vials and cans, while a relatively high seed moisture content of 8.32% for wheat and 8.49% for barley was reported. Atmospheric oxygen is known to reduce seed longevity (Ellis and Hong, 2007a; Groot *et al.*, 2015), while a seed moisture content of 3–7% is generally recommended for long-term storage (Rao *et al.*, 2006). Therefore, in comparison to the study of Stanwood, more optimal conditions applied to the seeds stored at CGN. Prior to storage at CGN, the three-layered aluminium foil seeds bags are hermitically closed by vacuum sealing. Seed samples of CGN are dried at 15°C and 15% RH for two months in a controlled environment, which is in line with recommended conditions to reduce the moisture content of seeds to ~5% (IBPGR, 1982). For wheat and barley seeds, assuming an oil content of 2.0–2.2%, the equilibrium moisture content is 6% when seeds are dried at conditions practiced at CGN (Royal Botanic Gardens Kew, 2017), falling within the recommended range of 3–7%. The relative humidity of CGN’s drying room is registered every 15 min, showing only small increases when the drying room is visited and a fast recovery afterwards. No prolonged periods of high relative humidity have ever been registered during seed drying at CGN. Although the moisture content of seeds is not tested directly at CGN prior to storage, it is considered highly unlikely that the rapid loss of seed viability observed at 4°C is due to a suboptimal seed moisture content. Firstly, previous experiments using 50 wheat and 50 barley seed samples that had been stored for at least 20 years at 4°C at CGN showed that seed weight before and after re-equilibration at 15°C and 15% RH were highly correlated (Groot *et al.*, 2015). Secondly, data on seed moisture content collected in the present study fell within the range that is recommended for long-term storage. Moreover, the observed reductions in germination were much more severe than could be expected based on the assessed eRH, albeit that these measurements were not performed on the same seed bags. However, the different seed bags used for germination testing and eRH measurements originated from the same seed batch and had experienced identical handling procedures and environmental conditions during genebank management. Therefore, a similar moisture content can be expected when the seeds were divided over different bags prior to storage.

The fact that the loss of viability varies among accessions of the same species (Table 1, Fig. 3) may indicate intra-specific variation in seed characteristics. Therefore, longevity estimates for a taxon based on single seed lots should be treated with caution. The finding that seed samples lose their viability more rapidly under 4°C in comparison with −20°C storage is in itself not particularly surprising as such temperature effects have already been demonstrated in artificial ageing studies (e.g. Dickie *et al.*, 1990). However, the fact that the effect was observed under actual and optimized genebank conditions, for crops that are generally considered good maintainers, and within considerably shorter time frames than generally expected, is considered rather alarming. For this reason CGN has decided to store also the active collection at −20°C. Although genebanks often maintain seed samples at 4°C only for medium-term storage of the active collection, the distribution of seeds with low viability should be regarded as a poor service to the user community. The findings of our study should be a real concern to genebanks that also store their base collection at suboptimal temperatures. In case storage at proper temperatures is not an option, regular monitoring of seed viability and timely rejuvenation of accessions should prevent the loss of valuable genetic resources contained in such collections.

## Supplementary Material

Supplementary Data
