# Supplementary material for: Rapid loss of seed viability in ex situ conserved wheat and barley at 4°C as compared to −20°C storage
Source: Conserv Physiol. 2018 Jun 25;6(1):coy033. doi: 10.1093/conphys/coy033 (PMC6016650; doi:10.1093/conphys/coy033)
Supplement: Supplementary Data [file coy033_van_treuren_et_al_conphys-2017-062_revision_3_suppl_data.docx]

**Supplementary Table 1:** CGN accession numbers of the study materials of wheat and barley per regeneration year. Underlined wheat accessions were retested in 2017.

| **Wheat** | | | | |  | **Barley** | | | | |
| --- | --- | --- | --- | --- | --- | --- | --- | --- | --- | --- |
| **1978** | **1979** | **1984** | **1985** | **1986** |  | **1985** | **1986** | **1987** | **1988** | **1989** |
| 12386 | 06033 | 05596 | 04040 | 05354 |  | 00007 | 00071 | 00521 | 01003 | 00985 |
| 12405 | 08537 | 06027 | 04043 | 05363 |  | 00008 | 00072 | 00524 | 01037 | 01051 |
| 12409 | 08538 | 06037 | 04054 | 05395 |  | 00019 | 00103 | 00549 | 01040 | 01119 |
| 12549 | 08539 | 06104 | 04075 | 05396 |  | 00032 | 00112 | 00826 | 01175 | 01153 |
| 12561 | 08557 | 06112 | 04123 | 05398 |  | 00044 | 00113 | 00829 | 01767 | 01207 |
| 12566 | 08764 | 06140 | 04125 | 05409 |  | 00045 | 00417 | 00850 | 01774 | 01634 |
| 12619 | 08766 | 06143 | 04136 | 05411 |  | 00096 | 00463 | 00855 | 01798 | 01686 |
| 12626 | 08818 | 06146 | 04142 | 05413 |  | 00138 | 00497 | 00859 | 01799 | 01728 |
| 12658 | 08836 | 06185 | 04150 | 05419 |  | 00139 | 01261 | 00885 | 01807 | 01776 |
| 12664 | 08874 | 06195 | 04166 | 05434 |  | 00149 | 01265 | 00897 | 01809 | 01806 |
| 12665 | 08875 | 06209 | 04167 | 05448 |  | 00151 | 01302 | 00902 | 01825 | 01823 |
| 12667 | 08890 | 06224 | 04169 | 05458 |  | 00157 | 01310 | 01616 | 01829 | 01831 |
| 12675 | 08899 | 06226 | 04175 | 05478 |  | 00241 | 01343 | 01628 | 02686 | 02318 |
| 12697 | 08904 | 06228 | 04184 | 05493 |  | 00287 | 01412 | 01639 | 02691 | 02547 |
| 12698 | 08913 | 06304 | 04232 | 05538 |  | 00290 | 01438 | 01641 | 02698 | 02557 |
| 12705 | 08919 | 06309 | 04252 | 05544 |  | 00294 | 01442 | 01666 | 02819 | 02576 |
| 12709 | 08952 | 06314 | 04284 | 05549 |  | 00297 | 01974 | 01681 | 11179 | 02710 |
| 12711 | 08953 | 06333 | 04307 | 05589 |  | 00337 | 02068 | 01692 | 11183 | 02719 |
| 12713 | 08963 | 06348 | 04311 | 05621 |  | 02589 | 02072 | 01706 | 11188 | 11294 |
| 12721 | 08976 | 06353 | 04326 | 05638 |  | 02608 | 02074 | 01711 | 11234 | 11301 |
| 12722 | 08997 | 06356 | 04336 | 05731 |  | 02613 | 02079 | 01715 | 11238 | 11863 |
| 12733 | 09004 | 06393 | 04372 | 05752 |  | 02615 | 02138 | 01734 | 11274 | 11866 |
| 12738 | 09060 | 06445 | 04420 | 07998 |  | 02619 | 02524 | 01737 | 11278 | 11867 |
| 12747 | 09132 | 06455 | 05357 | 08078 |  | 02621 | 02528 | 01746 | 11282 | 13021 |
| 12754 | 09267 | 06458 | 10397 | 08088 |  | 02638 | 03378 | 01757 | 11302 | 13025 |

**Supplementary Table 2**: Equilibrium relative humidity (eRH) at 15°C and percentage moisture content (%MC) of wheat and barley seeds stored at CGN at -20°C and 4°C, respectively. ‘Acc’ denotes the accession number and ‘Year’ the year in which the accession was regenerated. ‘G0’ indicates the fraction germination prior to storage and G1 the fraction germination tested for wheat in 2011 and for barley in 2012. Underlined scores refer to seed bags that showed incomplete vacuum when retrieved from the storage facility.

|  | | | **-20°C storage** | | | **4°C storage** | | | |
| --- | --- | --- | --- | --- | --- | --- | --- | --- | --- |
| **Acc** | **Year** | **G0** | **G1** | **eRH** | **%MC** | **G1** | **eRH** | **%MC** | |
| **Wheat** | | | | | | | | | |
| 12409 | 1978 | 0.99 | 0.98 | 20.7 |  | 0.77 | 22.2 | |  |
| 12722 | 1978 | 0.98 | 0.99 | 16.4 |  | 0.77 | 23.5 | |  |
| 9267 | 1979 | 0.94 | 0.97 | 15.4 |  | 0.36 | 16.0 | |  |
| 8539 | 1979 | 0.97 | 1.00 | 17.0 | 5.42 | 0.77 | 17.3 | |  |
| 6027 | 1984 | 0.96 | 0.96 | 14.6 |  | 0.86 | 18.3 | | 6.24 |
| 6455 | 1984 | 0.95 | 0.95 | 17.0 | 6.14 | 0.96 | 21.1 | | 5.93 |
| 4123 | 1985 | 0.96 | 0.91 | 15.0 | 5.97 | 0.29 | 16.8 | | 6.07 |
| 4232 | 1985 | 0.94 | 0.98 | 13.2 |  | 0.79 | 15.6 | |  |
| 5411 | 1986 | 0.94 | 0.98 | 13.1 |  | 0.49 | 28.7 | | 7.56 |
| 5752 | 1986 | 1.00 | 1.00 | 11.1 |  | 0.81 | 14.9 | |  |
| **Barley** | | | | | | | | | |
| 2613 | 1985 | 0.95 | 0.79 | 17.0 |  | 0.42 | 15.5 | |  |
| 157 | 1985 | 0.98 | 0.98 | 23.9 |  | 0.99 | 25.2 | |  |
| 113 | 1986 | 0.98 | 0.92 | 19.1 |  | 0.52 | 20.1 | |  |
| 2068 | 1986 | 0.99 | 1.00 | 14.2 |  | 0.98 | 16.3 | |  |
| 1692 | 1987 | 0.96 | 0.88 | 11.9 |  | 0.29 | 15.0 | |  |
| 859 | 1987 | 0.97 | 0.94 | 15.7 |  | 0.90 | 20.7 | |  |
| 11302 | 1988 | 0.82 | 0.85 | 13.7 | 5.69 | 0.32 | 22.5 | | 7.00 |
| 2698 | 1988 | 1.00 | 0.98 | 14.1 | 5.81 | 1.00 | 16.1 | | 5.52 |
| 1823 | 1989 | 0.92 | 0.88 | 22.2 |  | 0.77 | 33.6 | |  |
| 1806 | 1989 | 0.95 | 0.96 | 22.1 |  | 0.97 | 25.1 | |  |
